# Safety of Anesthesia With Dexmedetomidine for Muscle and Nerve Biopsy – A Review of 100 Cases

**DOI:** 10.7759/cureus.89859

**Published:** 2025-08-12

**Authors:** Satoko Uruha, Akinori Uruha, Tomoya Kawazoe, Masako Mukai, Nanae Miyake, Shiro Fukuda, Hiroaki Matayoshi, Keizo Sugaya, Kazushi Takahashi

**Affiliations:** 1 Department of Neurology, Tokyo Metropolitan Neurological Hospital, Tokyo, JPN; 2 Department of Medicine (Neurology and Rheumatology), Shinshu University School of Medicine, Matsumoto, JPN; 3 Department of Anesthesiology, Tokyo Metropolitan Neurological Hospital, Tokyo, JPN

**Keywords:** dexmedetomidine, muscle biopsy, myopathy, nerve biopsy, neuropathy

## Abstract

Pain during muscle and nerve biopsies is a distressing complication for patients. Dexmedetomidine, an α2-adrenoreceptor agonist, has analgesic as well as sedative effects and causes less respiratory depression. This study aimed to clarify the safety of anesthesia using dexmedetomidine for muscle and nerve biopsies. We retrospectively evaluated the safety in 100 patients who underwent anesthesia with dexmedetomidine during muscle or nerve biopsies at our institution. Adverse events were defined as follows: bradycardia as heart rate <50 bpm, hypotension as systolic blood pressure <70 mmHg, desaturation as SpO2 < or =93%, and delayed awakening as being unable to drink or eat within two hours post-operation. The major adverse events were bradycardia (20 out of 98 patients, 20%) and hypotension (six out of 100 patients, 6%); however, these adverse events did not interrupt the biopsy. There was no adverse desaturation. None of the biopsies had to be interrupted due to any other adverse event. Our findings suggest that dexmedetomidine can be safely administered for muscle and nerve biopsies.

## Introduction

Muscle biopsy plays a central role in the diagnostic practice and research of muscle diseases. However, pain during open muscle biopsy is often a distressing complication for patients. Less invasive methods, such as Duchenne’s harpoon, needle biopsies [1,2] and conchotome biopsies [3,4], have been introduced; but it is often difficult to obtain a sufficient sample of muscle tissue. Open biopsies under local anesthesia are still the most widely used method. However, it is necessary to avoid anesthetizing the muscle itself because the infiltration of local anesthetic into the muscle could cause significant artifacts such as fiber necrosis, edema, and inflammatory cell infiltration that could complicate or obscure diagnostic interpretation [2,3]. Therefore, pain when cutting the muscle inevitably occurs. To reduce this pain, non-opioid pain relievers or anti-anxiety medications are sometimes used in conjunction with local anesthesia; however, these treatments may not sufficiently alleviate it. This problem applies to nerve biopsies as well. Pain during muscle and nerve biopsies is a major concern in clinical practice due to the invasive nature of these procedures and the potential for postoperative complications.

Dexmedetomidine (DEX), an α2-adrenergic receptor agonist, is a common intravenous sedative agent [5]. DEX provides superior analgesia compared to other sedatives. Since it is intravenously administered, and not directly injected into the biopsy site, artifacts do not occur. Also, the risk of respiratory depression is low [5-8]. As patients with neuromuscular diseases often have respiratory impairment, we considered these features of DEX to be advantageous for muscle and nerve biopsies. However, it is known that bradycardia and hypotension can occur during anesthesia with DEX [9]. This study aimed to clarify the safety of anesthesia using DEX for muscle and nerve biopsies.

## Materials and methods

We conducted a retrospective analysis of 100 patients who underwent muscle or nerve biopsies under anesthesia with DEX at the Tokyo Metropolitan Neurological Hospital between 2019 and 2023. The cohort consisted of 63 muscle and 37 nerve biopsies. The median age was 64.5 (17-86) years, with an equal sex distribution. All patients received a prophylactic administration of continuous oxygen (1-3 L/min), except for one patient who received non-invasive ventilation. Two patients had a pacemaker implanted. The procedure for administering DEX was based on the package insert: a loading dose of 6 µg/kg/hr administration for the first 10 minutes and a maintenance dose of 0.2-0.7 µg/kg/hr (tolerated maximum dose to achieve sufficient sedative and analgesic effect, defined as -2 to -4 on the Richmond agitation-sedation scale). DEX was discontinued immediately after cutting the muscle or nerve. In all cases, local anesthesia was administered to the subcutaneous area using 1% lidocaine with 100,000 units of epinephrine. Adverse events were defined as follows: bradycardia as heart rate <50 bpm, hypotension as systolic blood pressure <70 mmHg, desaturation as SpO2 < or =93%, and delayed awakening as being unable to drink or eat within two hours post-operation. We defined these criteria as clinically significant low levels that are clearly troublesome in practice.

Based on the precautions in the package insert for DEX, patients at high risk, such as respiratory insufficiency, severe cardiac dysfunction, or marked autonomic symptoms, were excluded in advance under the judgment of the attending physician. Consequently, one patient with respiratory dysfunction and one with cardiac dysfunction were excluded. Pediatric patients aged 15 or younger were also excluded.

This study was approved by the institutional review board of the Tokyo Metropolitan Neurological Hospital (approval no. TS-R06-038).

## Results

All patients, whose clinical diagnoses are shown in Table 1, were at a level of anesthesia where they either did not respond or responded briefly before transitioning into a drowsy state.

**Table 1:** Clinical diagnosis of the subjects LGMD: Limb girdle muscular dystrophy; OPDM: Oculopharyngodistal myopathy; OPMD: Oculopharyngeal muscular dystrophy; FSHD: Facioscapulohumeral muscular dystrophy; CIDP: Chronic inflammatory demyelinating polyneuropathy; ALS: Amyotrophic lateral sclerosis; SCD: Spinocerebellar degeneration; MGUS: Monoclonal Gammopathy of Undetermined Significance; CMT: Charcot-Marie-Tooth disease.

Clinical diagnosis		Clinical diagnosis	
Mitochondrial diseases	11	CIDP	3
LGMD	4	Diabetic neuropathy	2
OPDM	2	Amyloidosis	2
OPMD	2	ALS	2
Centronuclear myopathy	1	SCD + neuropathy	2
Myofibrillar myopathy	1	IgG4-related disease	1
FSHD	1	MGUS + neuropathy	1
Myopathy (Undiagnosed or under investigation)	8	CMT	1
Sjögren syndrome	1	Toxic neuropathy	1
Paraneoplastic syndrome	1	Neuropathy (Undiagnosed or under investigation)	15

To maintain this depth of anesthesia, the maintenance dose of DEX was continued at the maximum dose in 98 of 100 patients (two patients presented with bradycardia or hypotension). Regarding the adverse events of anesthesia with DEX, bradycardia was observed in 20 patients (20 of 98 patients, 20%). In two cases, the patients’ heart rate dropped below 40 bpm but improved with a reduction in the dosage of DEX. For the other patients, hemodynamics remained stable, and the procedure was continued with careful monitoring. Hypotension occurred in six patients (out of the 100, 6%), four of whom required intervention (two patients required supplemental fluid administration, one required leg elevation, and one required DEX dose reduction), while none of the patients had to discontinue the biopsy. The median age of patients was 74.5 (36-80) years, which was higher than that of patients without hypotension; 64 (17-86) years, p=0.28, Mann-Whitney U Test (Table 2).

**Table 2:** Patients with hypotension MGUS: Monoclonal Gammopathy of Undetermined Significance.

Age (years)	Sex	Clinical diagnosis	Pre-biopsy blood pressure (mmHg)	Nadir blood pressure (mmHg)	Pre-biopsy heart rate (bpm)
80	Male	MGUS + neuropathy	119/73	62/31	55
78	Male	Myopathy	105/76	64/43	76
77	Male	Myositis	119/70	62/39	70
72	Female	Myopathy	143/63	63/31	63
59	Male	Mitochondrial diseases	111/67	69/42	67
36	Male	Neuropathy	109/83	61/32	83

Except for one, all were male patients. No association with any specific disease was observed. Delayed awakening was recorded in eight patients. None required additional treatment, and all patients eventually woke without complications within a few hours. Only one case of hypertension was recorded, where the patient's blood pressure rose from 120 mmHg pre-biopsy to 173 mmHg after the 10-minute induction dose of DEX. The patient’s blood pressure gradually decreased to 130 mmHg without any special intervention. There were no cases of oxygen desaturation. None of the biopsies had to be interrupted due to any other adverse event.

## Discussion

All 100 patients completed the biopsy under DEX. The major adverse events were bradycardia and hypotension (20% and 6%, respectively). In the clinical setting, hypotension requires particular caution. The older patients were more likely to develop hypotension in our investigation, although no statistically significant difference was found. A study reported older age as a risk factor linked to hemodynamic instability during DEX administration in intensive care unit patients [10]. Cardiac adverse events were more frequent in patients who had cardiac diseases, electrocardiography abnormalities, and those who used DEX in combination with other anesthetics [11-15]. In this study, an association between cardiac adverse events and comorbidity with cardiac diseases was not observed (there was no significant comorbidity of cardiac diseases). Furthermore, we did not administer DEX combined with other intravenous anesthetics, except for local lidocaine anesthesia. Previous studies reported that DEX can cause hypertension with rapid intravenous infusion or a single rapid dose because it constricts peripheral blood vessels [9,16]. This phenomenon was transiently observed in one patient.

No patient experienced significant desaturation, although minimal oxygen administration was provided, which may have contributed to this finding. This suggests that DEX offers greater benefits compared with other sedative agents, particularly for patients with neuromuscular diseases, who are more prone to respiratory impairment. This has important implications in clinical settings.

Administration of DEX is easy to perform. Although nerve blocks for muscle and nerve biopsies have been occasionally used, they require proficiency in the technique. On the other hand, anesthesia with DEX is simple and straightforward, involving basic intravenous administration without any special technique. Also, since it is intravenously administered (not directly injected into the biopsy site), artifacts do not occur.

This study has several limitations. First, we did not use DEX for high-risk patients, such as those with compromised respiratory function, those with severe heart failure, and those with strong autonomic symptoms. This might introduce selection bias; however, since only two patients were preemptively excluded, the influence of bias will be minimal, if present. Second, this study enrolled adult patients only and did not evaluate pediatric patients. As sedation is more often necessary for pediatric patients, this warrants further investigation. Finally, the efficacy of pain reduction was not objectively assessed since this study focused on the safety of DEX in muscle and nerve biopsies. Patients who undergo biopsies with conventional treatment using only local anesthesia usually report severe pain; however, those who received anesthesia with DEX did not complain of severe pain and reported no recollection of pain following biopsy. Since this study focuses just on the safety of DEX, we did not consider the inclusion of a control group necessary. Future comparative studies are necessary to demonstrate that anesthesia with DEX is a suitable method of pain relief during muscle and nerve biopsies.

## Conclusions

Our findings suggest that anesthesia with DEX is a safe option for muscle and nerve biopsies. Particularly, there was no recorded significant desaturation. This fact will strengthen the advantage of DEX for individuals with neuromuscular diseases, which are often associated with respiratory impairment. Moreover, its simple administration method makes it suitable for use in a wide range of healthcare settings.

However, since cardiac adverse events, such as bradycardia and hypotension, can occur, albeit rarely, close monitoring is essential to ensure patient safety during the procedure, especially for older patients. Additionally, though muscle and nerve biopsies under anesthesia with DEX will alleviate pain, further studies are necessary to demonstrate its efficacy.
